# Evaluation of mitral valve parameters with gender and age in the Pakistani population: A 2-dimensional echocardiographic study

**DOI:** 10.12669/pjms.42.9.13469

**Published:** 2026-09

**Authors:** Mahail Khan, Aisha Qamar, Sabha Bhatti, Aneel Ahmed

**Affiliations:** 1 Mahail Khan, MBBS, MPhilAssistant Professor, Department of Anatomy, Bahria University Health Sciences Campus Karachi (BUHSCK), Karachi, Pakistan; 2 Aisha Qamar, MBBS, MPhilSenior Professor, Department of Anatomy, Bahria University Health Sciences Campus Karachi (BUHSCK), Karachi, Pakistan; 3 Sabha Bhatti, MBBS, Internal Medicine Residency, Cardiology Fellowship, Followed by Advance Cardiac Imaging Fellowship with Level III training in Echocardiography, Nuclear cardiology/PET, Cardiac CT, Cardiac MR imaging and Structural Interventional Cardiology Imaging. Professor, Director & Fellowship Program Director Cardiac Imaging, National Institute of Cardiovascular Diseases (NICVD), Karachi, Pakistan; 4 Aneel Ahmed, MBBS, FCPS & Fellowship in Advance Cardiac Imaging Senior Registrar, Sindh Institute of Cardiovascular Diseases (SICVD), Sukkur, Pakistan

**Keywords:** Age Factors, Echocardiography, Mitral Valve, Sex Factors, Pakistan

## Abstract

**Objectives:**

This study aimed to assess variations in mitral valve echocardiographic parameters in relation to gender and age using transthoracic 2-dimensional echocardiography in a subset of the Pakistani population.

**Methodology:**

This cross-sectional study was conducted over six months (March–August 2024) at the National Institute of Cardiovascular Diseases (NICVD), Karachi, enrolling 385 individuals who fulfilled the inclusion criteria. Mitral valve echocardiographic parameters were measured using transthoracic 2D echocardiography and recorded on subject evaluation forms. Variations across gender and four age groups (18–30, 31–45, 46–65, and >65 years) were analyzed using the Kolmogorov-Smirnov test, Shapiro-Wilk test, Mann-Whitney U test, and Kruskal-Wallis test.

**Results:**

Among 385 participants, 182 males and 203 females were examined. Posterior leaflet length was significantly greater in males than in females (p = 0.012), while all other parameters — annular diameter, annular area, anterior leaflet length and thickness, and posterior leaflet thickness — showed no statistically significant difference between genders. No parameter showed a statistically significant difference across age groups; posterior leaflet thickness did not reach statistical significance across age groups (p = 0.069).

**Conclusion:**

In the Pakistani population, posterior leaflet length is significantly greater in males than in females. No statistically significant age-related differences were observed across any mitral valve parameter. These findings indicate a need for gender-specific reference values in echocardiographic assessment of the mitral valve in this population.

## INTRODUCTION

The mitral valve (MV) is a dynamic cardiac structure that ensures unidirectional blood flow during ventricular systole. It comprises an annulus, two leaflets, chordae tendineae, and papillary muscles, whose coordinated function prevents retrograde flow into the left atrium; structural incompetence can lead to significant morbidity and mortality.^1^ Unlike other cardiac valves, MV differs mainly in structure and composition, having a non-uniform surface due to the bundle of fibers, which are large, visible, and running in all directions, so that the thickness of the mitral valve varies across the surface, which not only differs on the same valve but also between humans and even entities of similar species.^2^ Due to the rising awareness, gender and age have a marked impact on cardiac morphology, disease mechanisms, and treatment outcomes. It is now crucial for researchers to understand the morphological variation of this complex construct across various demographic groups.^3^,^4^ Developments in cardiac imaging and operative approaches have established that MV configuration demonstrates significant variations exceeding mere dimensional distinctions, influencing clinical diagnosis, preoperative planning, and long-term outcome estimation.^5^

### Gender-based variations:

The World Alliance Societies of Echocardiography (WASE) study highlighted this demographic heterogeneity. It accentuated the need for normative data specific to the population to prevent diagnostic errors and redundant medical treatments. The research analyzed that even after adjusting for heart size in healthy individuals, men showed larger dimensions of various parameters of MV.^6^ However, a higher incidence of mitral valve disease like bileaflet prolapse associated with interstitial fibrosis is more specific to women. This gender-based difference has been accredited to biological factors, incorporating disparities in myocardial fibrotic remodeling and hormonal influences, rather than solely to the hemodynamic severity like mitral regurgitation or mitral annular disjunction.^7^

### Age-related variations:

The MV endures notable anatomical modifications throughout the human lifetime. The study on children even suggests deviations in MV annular geometry as they grow^8^, while significant changes occur in cardiac morphology in individuals over 80 years old.^9^ These alterations with age occur in unification with progressive changes in the MV parameters, which encompass an increase in the thickness of the leaflet with fibrotic changes.^10^ This results in a larger annular area, increased leaflet surface area, and reformed thickness due to adaptive remodeling of the MV with advancing age.^11^ With the advancement in minimally invasive MV interventions, it has become crucial to understand the anatomical variations for preoperative strategy and therapeutic outcomes of MV repair or prosthetic valve replacement according to gender and age.

Although worldwide evidence on MV morphological measurements is emerging, local data from Pakistan remains insufficient. Gurmani et al.^12^ validated that echocardiographic parameters contrast between the Pakistani population and Western reference values, yet standardized, normative MV data according to gender and age specific to Pakistan is limited. Without locally derived baseline values, clinicians may incorrectly identify normal morphological variants as pathological or vice versa, leading to diagnostic errors and potentially redundant or delayed interventions. This gap is particularly significant as Pakistan has one of the highest prevalence of rheumatic heart disease globally, where MV is the most affected anatomical structure.

Current standards for assessing mitral valve dimensions are predominantly based on data from Western populations, which may not apply to diverse demographic settings in Pakistan. Thus, this study aims to assess variations in mitral valve echocardiographic parameters in relation to gender and age using transthoracic 2-dimensional echocardiography in a subset of the Pakistani population.

## METHODOLOGY

This cross-sectional study was conducted over six months, from March to August 2024, involving 385 individuals, including both males and females, with ages ranging between 18 and 75 years. Non-probability consecutive sampling was employed, which was conducted at the National Institute of Cardiovascular Diseases (NICVD) in Karachi. All eligible participants were consecutively enrolled who met the predefined inclusion and exclusion criteria during the study period, without selective recruitment. Furthermore, all participants underwent a standardized transthoracic echocardiographic assessment using a uniform imaging protocol, ensuring consistency in data collection. The sample size of 385 was calculated based on the population size of 1,000,000. For this estimation, a maximum variation of 50% with a 95% confidence interval and a ±5% margin of error was considered to ensure statistical significance and generalizability. This was determined using the open-source calculator available at www.openepi.com, version 3.01-SSPropor.

### Ethical approval:

This study has been approved by the Institutional Review Board Committee of Bahria University Health Sciences Campus Karachi (BUHS-IRB# 0320/23-new-24; dated February 2, 2024) and the National Institute of Cardiovascular Diseases, Karachi (NICVD) (Ref# IRB-18/2024; dated February 28, 2024). The study parameters and their rationale were explained to each participant in either English or Urdu by the principal investigator, and then informed consent was obtained voluntarily. and participants were informed during the consent process that they could withdraw from the study at any time without providing a reason. No monetary or other incentives were provided to participants.

### Inclusion & Exclusion criteria:

Both males and females aged between 18 to 75 years were included in the study. Eligibility was evaluated through a combination of medical history, clinical records, participant interview, physical examination, and transthoracic echocardiographic findings. Participants with any history of previous mitral valve surgery and intervention, known structural abnormalities (like mitral valve prolapse, rheumatic heart disease, or congenital mitral valve malformation), pregnant women, athletes, physical limitations (like severe obesity or chest wall deformation), or poor imaging by 2D Transthoracic Echocardiography were excluded from the study. These exclusion criteria were applied to reduce confounding factors by conditions linked with physiological or pathological cardiac remodeling, thereby permitting a more precise evaluation of MV parameters in the study population.

### Imaging technique:

The height and weight of the individuals were measured by using a calibrated mechanical physician beam balance scale with an attached stadiometer available in the echocardiography department. For capturing cardiac measurement, Cannon Aplio i600 CV echocardiography machine, frequency range 1-22 Mhz, iBeam, ApliPure+, CEUS: Quad view, Display modes 2D, 3D was used. Echocardiographic examinations were performed by two experienced cardiac sonographers. All measurements were reviewed and verified by a consultant cardiologist under the supervision of a consultant cardiac imaging specialist. To minimize observer variability, a standardized imaging protocol with predefined anatomical landmarks and uniform measurement criteria was followed throughout the study. Although formal interobserver and intra-observer variability analyses were not performed, adherence to a standardized protocol and expert review was intended to enhance measurement consistency. The study’s parameters were all recorded on the subject evaluation form. The annular area was calculated by the formula: (MA area = d1 x d2 x π/4) where d1is annular diameter (long-axis view) and d2 is annular diameter (4-chamber view).^13^ All parameters were measured in the end diastolic phase when the mitral valve is relaxed and most open, providing the most constant reference point. Most of the measurements were obtained from a parasternal long-axis (PLAX) view, which included the annular diameter (long-axis), length, and thickness of the mitral valve leaflets. Only the annular diameter was measured in the 4-chamber view of the echocardiography (Fig.1).

**Fig.1:**
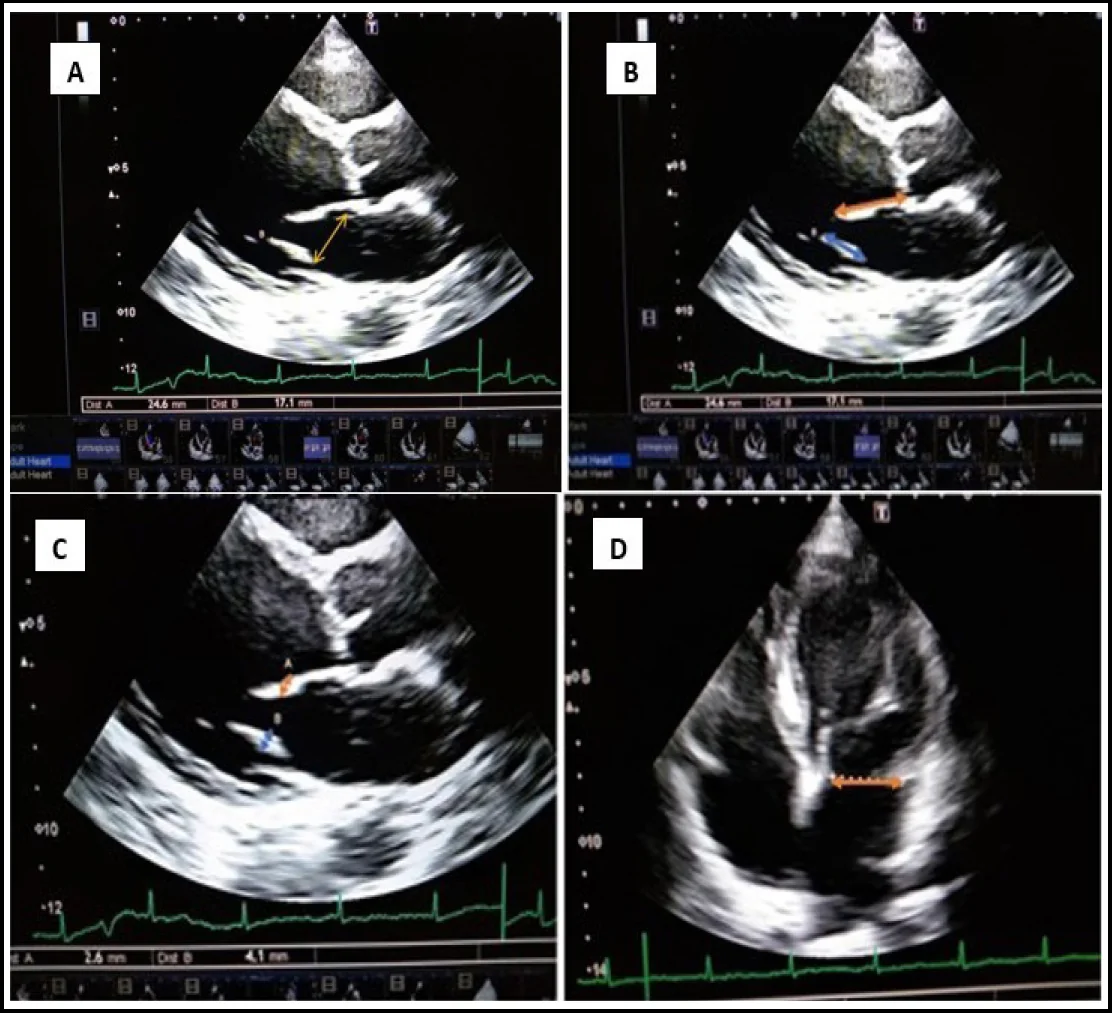
Echocardiographic image measuring in the PLAX view (A) an annular diameter (B) anterior (orange arrow) and posterior (blue arrow) leaflet length (C) anterior (orange arrow) and posterior (blue arrow) leaflet thickness (D) Echocardiographic image measuring an annular diameter of the mitral valve in a 4-chamber view from the current study conducted at NICVD, Karachi.

### Statistical analysis:

SPSS version 23.0 was used to analyze all the data. To check the normality of the data, Kolmogorov-Smirnov and Shapiro-Wilk tests were applied. Due to the non-normality of the data, non-parametric tests, the Mann-Whitney U test and Kruskal-Wallis test were applied to compare two independent and more than two independent variables, respectively. The level of statistical significance (p-value) was set at < 0.05.

## RESULTS

The selected sample size was 385 in total, comprising of 182 male (47%) and 203 female (53%) and were classified into four groups according to age:18-30 years (young adults), 31-45 years (middle-aged adults), 46–65 years (older adults), and >65 years (elderly), as seen in Fig.2.

**Fig.2:**
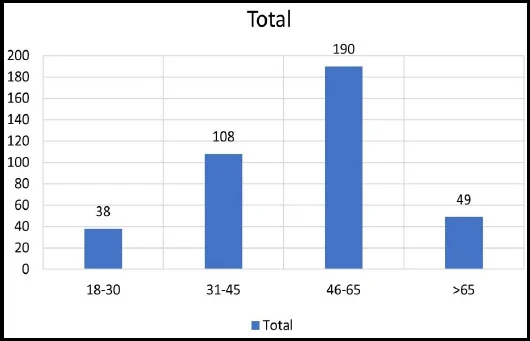
Distribution of study participants into age group (in years).

In the current study, all mitral valve echocardiographic parameters were compared between genders and various age groups. These comparisons were demonstrated in ranks, and the sum of ranks as the non-parametric tests were applied. In Table-I, the Mann-Whitney U test was applied to compare MV parameters between male and female participants. All parameters, including annular diameter (both in PLAX and 4-chamber view), annular area, anterior leaflet length and thickness, and posterior leaflet thickness, showed no statistically significant difference between males and females, except for posterior leaflet length, that showed statistically significant higher mean rank in males than in females, suggesting male having longer posterior MV leaflet than female.

**Table-I:** Measurement of echocardiographic parameters (Based on Gender).

Parameters Gender		N (385)	Mean Rank	Sum of Ranks	p-value
Annular diameter long axis	Male	182	201.49	36671.50	0.156
	Female	203	185.39	37633.50	
Annular diameter four chamber	Male	182	197.51	35947.00	0.451
	Female	203	188.96	38358.00	
Annular area	Male	182	197.40	35926.00	0.463
	Female	203	189.06	38379.00	
Anterior leaflet length	Male	182	200.22	36440.00	0.227
	Female	203	186.53	37865.00	
Anterior leaflet thickness	Male	182	196.18	35705.00	0.593
	Female	203	190.15	38600.00	
Posterior leaflet length	Male	182	207.94	37845.00	0.012*
	Female	203	179.61	36460.00	
Posterior leaflet thickness	Male	182	192.63	35058.00	0.950
	Female	203	193.33	39247.00	

In Table-II, the Kruskal-Wallis test was applied to compare MV parameters across the four age groups. No statistically significant age-related variations were observed in any of the measured mitral valve parameter. Posterior leaflet thickness demonstrated higher values in the older age groups; however, this difference also did not reach statistical significance (p = 0.069).

**Table-II:** Measurement of echocardiographic parameters (Based on Age in years).

Parameters	Age	N	Mean Rank	p-value
Annular diameter long axis	18-30	38	154.62	0.113
	31-45	108	197.55	
	46-65	190	200.59	
	>65	49	183.31	
Annular diameter 4-chamber	18-30	38	172.07	0.314
	31-45	108	201.69	
	46-65	190	196.92	
	>65	49	174.86	
Annular area	18-30	38	160.91	0.142
	31-45	108	203.57	
	46-65	190	197.51	
	>65	49	177.11	
Anterior leaflet length	18-30	38	199.61	0.840
	31-45	108	198.48	
	46-65	190	191.12	
	>65	49	183.09	
Anterior leaflet thickness	18-30	38	180.87	0.885
	31-45	108	192.41	
	46-65	190	194.07	
	>65	49	199.55	
Posterior leaflet length	18-30	38	174.17	0.479
	31-45	108	194.92	
	46-65	190	199.23	
	>65	49	179.19	
Posterior leaflet thickness	18-30	38	149.91	0.069
	31-45	108	190.22	
	46-65	190	200.85	
	>65	49	202.12	

p-value ≤ 0.05: significant (*); Test applied: Mann-Whitney Test.

p-value ≤ 0.05: significant (*); Test applied: Kruskal-Wallis Test.

## DISCUSSION

The current study aimed to elucidate the mitral valve morphology among a representative sample of the Pakistani population and examined their variation according to gender and age. Our findings showed a significant variation in the posterior leaflet length between genders, with male having a larger posterior MV leaflet than female but no statistically significant age-related differences were classified.

The variation among gender coincides with previous studies that indicated gender-related dimorphic traits in MV anatomy. The WASE studied MV morphology in a large cohort of 748 healthy individuals using 3D transthoracic echocardiography and testified that, as compared to women, men had larger leaflet lengths, along with annular diameters and areas.^6^ Even though the WASE study utilized 3D echocardiography, the present study used 2D echocardiography, the two modalities are not directly comparable — 3D echocardiography permits more detailed assessment of the saddle-shaped mitral annulus and leaflet surface morphology, whereas 2D echocardiography evaluates linear measurements in predefined imaging planes. Despite this, the similr trends of our findings with the WASE study provides supportive validation. The perceived agreement may also redirect the use of standardized echocardiographic acquisition and measurement protocols in both studies, allowing reliable assessment of mitral valve morphology despite differences in imaging modality.

In contrast to our documentation regarding mitral annular diameters, Ricci et al. confirmed variations related to gender by having larger annular diameters in men using Cardiac Magnetic Resonance (CMR).^14^ Similarly, Naoum et al. examined mitral annular diameters using Cardiac Computed Tomography (CCT), which also showed extensive variation, with men having larger diameters.^15^ This divergence may be elucidated by distinctions in imaging modalities as CMR and CCT postulates high spatial resolution and may measure annular geometry contrarily from 2D echocardiography. Also, measurement procedures, study populations, and demographic characteristics may contribute to the differences. Even Savalgi et al. conducted a study on 50 embalmed human hearts aged between 20 to 60 years and reported men having a significant larger MV diameter and a slight increase in the width of the posterior leaflet in females.^16^ Although these findings differ from ours, the two studies are not directly comparable as Savalgi et al.^16^ measured in embalmed hearts, whereas we measured in living participants using echocardiography. In addition, embalming can modify tissue size and shape, which may affect valve quantification. Also, variances in the study populations (South Indians differ anthropometrically and ethnically from Pakistani population) and quantification protocols may have contributed to the distinction in findings. Therefore, these dissimilarities should be interpreted carefully rather than as contradictory results.

Another study conducted by Ring et al. assessed normal values for MV parameters using 3D transesophageal echocardiography (TEE) and documented variation in MV area, as men had larger mitral valve diameter and area, but showed no significance when the measurements were adjusted for body surface area, suggesting differences merely due to body size rather than inherent sexual differences.^17^ However, our findings showed a veritable anatomical difference, which is beyond the body size adjustment. This variation gives evidence of anatomical difference among both gender on cardiac connective tissue and remodeling. Although surgical outcomes were not assessed in the current study, previous studies showed females having more postoperative complications, making evident that clinical appearance and prognosis are influenced by the anatomical variations in MV. But despite this fact, females are less frequently referred for prompt operative procedures as compared to men.^18^,^19^ This discrepancy indicates the likelihood of not considering the gender related anatomical modifications following the existing diagnostic criteria and treatment strategies.

Hence, our findings suggest that morphological variations are selective with respect to gender, rather than being consistent across all parameters. This is supported by the observation that other parameters, such as annular diameter and area, anterior leaflet length and thickness, and posterior leaflet thickness, showed no significant variation. Thus, it is clinically essential to measure the posterior leaflet length precisely for surgical valve repair and replacement strategies.

Age-specific variations were not observed in our study, although the posterior leaflet thickness approached significance and displayed a thicker leaflet in the older age group, suggesting degenerative modifications such as fibrosis. The WASE study also measured MV parameters in various age groups and observed a significant increase in the dimensions with increasing age.^6^ Similarly, Gumpangseth et al. conducted research on 60 fresh embalmed hearts between 20 to 90 years of age in Thailand that showcased a statistically significant moderate positive correlation between age and length of leaflets of the MV that tends to increase with increasing age, unlike the current study.^20^ In addition to the difference in study design and tissue changes linked with embalming, this discrepancy may be due to the recruitment of more older participants (upto 90 years) and population specific demographic differences between Thai and Pakistanis.

Some studies have even documented gender and age-related MV variations in different phases of the cardiac cycle, such as Nemes et al. in the MAGYAR-Health study recorded greater annular dimensions in individuals above 50 years than in younger people in the end-systolic phase, while no differences were found during the end-diastolic using three-dimensional speckle-tracking echocardiography. Similarly, annular diameter, area, and perimeter measured at the end-diastolic phase had no statistically significant results, but it highlighted the significant difference at the end-systolic phase for gender as well, with females having smaller dimensions.^21^ This showed that significantly distinct variations are often seen during the systolic phase and might be specific to the age, due to a decrease in the contraction of the annulus with advancing age.

This study boosts the knowledge of mitral valve anatomy by highlighting the influence of demographic factors such as gender and age on its geometry. Although previous local studies predominantly focused on routine echocardiographic measurements but the current study, based on the available literature, is among the earliest to thoroughly assess MV morphology based on gender and age using 2D echocardiography in relatively large sample of participants. Additionally, the use of a standardized imaging protocol and expert analysis of echocardiographic measurements strengthen the reliability and uniformity of measurement method. The findings provide baseline data that may support future multicenter studies aimed at developing population-specific reference values, as it focuses on the importance of gender and age specific reference value in echocardiographic assessments. Additionally, Gurmani et al. established the difference in the echocardiographic measurements of cardiac parameters between the Pakistani population and Western reference standards,^12^ endorsing on requisite for localized reference values especially according to gender and age.

### Limitations:

It was a single-center and short-term study; therefore, the results cannot be generalized. Furthermore, mitral valve measurements were attained at a single phase of the cardiac cycle (end-diastole). As mitral valve geometry varies dynamically throughout the cardiac cycle, measurements at a single time point may not fully redirect to its functional morphology.

## CONCLUSION

This study highlights significant variation in echocardiographic measurements influenced by gender in the Pakistani population, with males exhibiting longer posterior mitral leaflets than females, while no statistically significant age-related differences were observed. This emphasizes the importance of establishing baseline values specific to our demographic factors rather than following the standardized measurements specific to the Western population and underscores the need to consider these factors in cardiovascular assessments and management.

### Future recommendations:

Future multicenter studies with larger sample sizes should employ advanced imaging modalities, such as three-dimensional echocardiography, CCT, or CMR for enhanced evaluation across multiple centers all over Pakistan for a longer period to elucidate the age-related trends seen in the MV parameters.
